# Biopsy Diagnosis of Oral Carcinoma by the Combination of Morphological and Spectral Methods Based on Embedded Relay Lens Microscopic Hyperspectral Imaging System

**DOI:** 10.1007/s40846-015-0052-5

**Published:** 2015-08-11

**Authors:** Mang Ou-Yang, Yao-Fang Hsieh, Cheng-Chung Lee

**Affiliations:** Department of Electrical and Computer Engineering, National Chiao-Tung University, 1001 Ta-Hsueh Rd., Hsinchu, Taiwan; Department of Optics and Photonics, National Central University, 300 Jhongda Rd., Jhongli, Taoyuan Taiwan

**Keywords:** Oral carcinoma, Hyperspectral imaging, Biopsy, Microscopy

## Abstract

Cytopathological examination through biopsy is very important for carcinoma detection. The embedded relay lens microscopic hyperspectral imaging system (ERL-MHIS) provides a morphological image of a biopsy sample and the spectrum of each pixel in the image simultaneously. Based on the ERL-MHIS, this work develops morphological and spectral methods to diagnose oral carcinoma biopsy. In morphological discrimination, the fractal dimension method is applied to differentiate between normal and abnormal tissues. In spectral identification, normal and cancerous cells are distinguished using five methods. However, the spectra of normal and cancerous cells vary with patient. The diagnostic performances of the five methods are thus not ideal. Hence, the proposed cocktail approach is used to determine the effectiveness of the spectral methods in correlating with the sampling conditions. And then we use a combination of effective spectral methods according to the sample conditions for diagnosing a sample. A total of 68 biopsies from 34 patients are analyzed using the ERL-MHIS. The results demonstrate a sensitivity of 90 ± 4.53 % and a specificity of 87.8 ± 5.21 %. Furthermore, in our survey, this system is the first time utilized to study oral carcinoma biopsies.

## Introduction

**Fig. 1:**
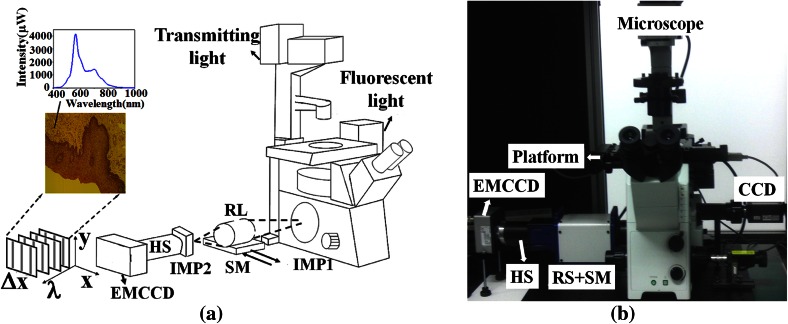
**a** Diagram and **b** photograph of ERL-MHIS (*RL* relay lens, *HS* hyperspectrometer, *SM* stepping motor, *IMP1* imaging plane 1, *IMP2* imaging plane 2, *FW* fluorescent wheel

Oral carcinoma is an important global health problem, as evidenced by approximately 40,250 new cases of oral and throat carcinoma detected in 2012 (American Cancer Society). In the United States, the overall 5-year survival rate for all stages of oral carcinoma is 61 % [1, 2]. Biopsy is the conventional means of diagnosing oral carcinoma [3, 4]. In addition to allowing pathologists to diagnose the stage of carcinoma accurately, biopsy also allows them to prescribe the most appropriate treatment. However, differences in experience and subjectivity when evaluating borderline dysplastic cells between pathologists might affect their diagnostic accuracy. A microscopic hyperspectral imaging system (MHIS) capable of presenting a tissue image and the spectral information of each pixel in the image simultaneously was developed to facilitate carcinoma diagnosis quantitatively [5–16]. However, both the hardware and analytical algorithm aspects of the conventional MHIS required further improvement. Regarding hardware, the conventional MHIS was time-consuming, had a complex mechanical structure, high off-axial optical aberration, and inconvenient alignment. The embedded relay lens MHIS (ERL-MHIS, Fig. 1) developed in our previous works overcomes these limitations [17, 18].

Certain limitations of analytical algorithms have prevented widespread MHIS application in carcinoma diagnosis [11–15]. Siddiqi et al. [13] distinguished between normal and cancerous cells by using the nuclear spectrum and then further distinguished them based on the nuclear/cytoplasmic ratio. Other works [11, 12, 14, 15] demonstrated that the nuclear spectrum can present quantitative differences between normal, precancerous, and cancer cells. However, there are several problems with these studies. First, these works used biopsies from only one patient. Although a 40 × objective was used to examine every single cell in the biopsy, their conclusions may not be applicable to all cancer patients. Second, the examination of every single cell in the biopsy was quite time-consuming. Third, most pathologists use a 20 × objective [19]. Accordingly, the use of a high-power objective (higher than 40×) is inconvenient for pathologists. Fourth, in a previous work, the sensitivity, specificity, or both of discrimination between normal and precancerous cells was about 70–80 % [13].

This work develops morphological and spectral methods and uses them to help pathologists quantitatively diagnose oral carcinoma biopsy. The proposed methods were used to diagnose 68 oral carcinoma biopsies of 34 patients using ERL-MHIS (with a 20 × objective). In the spatial domain, although the fractal dimension algorithm [20] can distinguish between the morphological differences of normal and abnormal tissues, an abnormal tissue does not represent a cancerous tissue. Hence, normal and cancerous cells in the tissue are distinguished in the spectral domain using five methods. However, the spectra of normal and cancerous cells vary with patient due to differences in sampling conditions (e.g., age of patients, lesion site, tumor size, and lymph node metastasis) [15, 21, 22]. Therefore, this work develops a novel cocktail approach to reduce the difference in the cell spectrum between patients. The proposed cocktail approach determines the effectiveness of spectral methods in correlating with the sampling conditions. The sample is then diagnosed using the optimal combination of effective spectral methods.

## Materials and Methods

### ERL-MHIS

Figure 1(a) illustrates the functions and setup of the ERL-MHIS. The designed relay lens for scanning is placed between the microscope and the hyperspectrometer. The stepping motor is located under the relay lens. The relay lens comprises symmetric infinite conjugate lenses for scanning and transferring images with optimal off-axis optical aberration. The object on the platform is imaged with the objective lens on imaging plane 1. The relay lens then transfers the image from imaging plane 1 to imaging plane 2, where the hyperspectrometer slit is located. The slit is along the y axis direction. Imaging plane 2 images one line (slit size) at a time on the electron-multiplying charged-coupled device (EMCCD). When the relay lens is static, the line image of slit size and the spectrum can be recorded on the EMCCD. While the stepping motor moves along the x-axis, the individual line images are recorded on the y–λ plane of the EMCCD. The stepping motor moves one step in the x-axis direction to capture the next line image and its spectrum. Each y–λ image is recorded as a single y–λ file for each row along the object corresponding to the radiation collection region, which maps through the hyperspectrometer to the EMCCD. After all of the line images are captured, the data cube of all of the y–λ files are loaded into memory.

The ERL-MHIS provides transmission and fluorescence images of the biopsy to assist pathologists in detecting cancerous cells or tissues. The transmission light source is a 100-W halogen lamp. The fluorescent light source is a 75-W xenon lamp. The transmission image provides morphological information and spectral information from 400 to 1000 nm of the cell or tissue. The fluorescence image provides the characteristic spectrum of the cell or tissue. The proposed system has two fluorescence modes (F1: 330–385 nm; F2: 470–490 nm). The fluorescence mode can be changed by tuning the fluorescent wheel. Figure 1(b) shows the finished product of the proposed ERL-MHIS, which consists of an inverted microscope (Olympus IX71), charged-coupled device (CCD; AVT PIKE F-421-C), RL, stepping motor (Sigma Koki, SGSP20-20), hyperspectrometer (Specim V10E, with spectral range of 400 to 1000 nm), and EMCCD (Andor Luca R604, with 1000 × 1000 pixels and 8-µm pixel size). The spatial resolution of the ERL-MHIS is 30 µm × 10 µm. The objective power affects the spatial resolution. This work uses a 20 × objective (spatial resolution: 1.5 µm × 0.5 µm). The spectral resolution of the ERL-MHIS is about 2.8 nm. The software for acquiring images and analyzing spectral information was programmed in the C language. The software controls the speed of the stepping motor, gain, and exposure time of the EMCCD.

### Patient Biopsy Preparations

Sixty eight biopsies of 34 oral carcinoma patients were provided by the China Medical University Hospital (Tai-Chung City, Taiwan). The 68 biopsies were divided into 58 training cases and 10 test cases. The type of test biopsy (normal or cancerous) was not revealed to the analyst who analyzed the 10 biopsies. The 58 training cases were utilized to determine the most effective methods for each sampling condition and its cut-off point. The performance of the proposed approach was validated using the 10 test cases.

Before the experiment, institutional review board (IRB) approval was obtained from China Medical University Hospital (IRB number DMR98-IRB-209). All patients received complete information on this experiment before providing their signed informed consent. This study was implemented in accordance with the Declaration of Helsinki.

The routine pathological diagnosis procedure with hematoxylin and eosin (H&E) staining was utilized to prepare the biopsies. After surgical operations, the oral carcinoma and normal samples were resected from the patients. Samples were then stained with H&E. Next, two biopsies (normal and cancerous) were prepared from each patient. The pathologist identified the biopsies as either normal or cancerous. Moreover, the pathologist marked the layers of the oral tissue (lamina propria and basal-cell layer) on the image of the biopsies. For each biopsy, one transmission image and two fluorescence images were acquired using the ERL-MHIS.

### Morphology-Based Fractal Dimension Method for Tissue Discrimination

Fractal dimension is a ratio that gives statistics of complexity, comparing how much detail in a pattern changes with the scale at which it is measured [20]. In this work, the complexity of the border between the lamina propria and the basal-cell layer is represented using the fractal dimension of the tissue image. Fractal dimension can be calculated by taking the limit of the quotient of the log change in object size and the log change in measurement scale, as the measurement scale approaches zero:

1 $$D = \frac{\log N}{\log s},$$

where *D* denotes the fractal dimension, *s* denotes the length of the chosen smallest unit, and *N* denotes the number of *s* required to cover the pattern. In this work, the nuclear size with nine pixels is used as the smallest unit. *N* is the image size (1000 × 1000 pixels). The fractal dimension is calculated using the binary image version of the transmission image.

The raw data of the transmission image must be calibrated before the fractal dimension is calculated. In this work, the dark noise of the system is removed using a dark image with no illumination. The nonuniformity of the transmission image is then removed using a reference blank, for which an area on the slide is scanned with all layers of glass besides the cell structures. The nonuniformity is caused by uneven illumination, scan line stripping, effect of the lamp, and reflectance or transmittance of glass. The calibration formula is:

2 $$T(\lambda ) = \frac{{I_{T} (\lambda ) - I_{dark} (\lambda )}}{{I_{white} (\lambda ) - I_{dark} (\lambda )}},$$

where *T(λ)* denotes the calculated transmittance value of each pixel in the transmission image, *I*_*T*_*(λ)* denotes the spectral intensity of raw data for each pixel in the transmission image, *I*_*dark*_*(λ)* denotes the spectral intensity of each pixel in the dark field, and *I*_*white*_*(λ)* denotes the spectral intensity of each pixel in the bright field.

Additionally, an attempt was made to acquire a binary image with a clear border between the basal-cell layer and the lamina propria by superimposing the transmission image in the wavelength range of 500–700 nm. The largest difference of spectral intensity between the basal-cell layer and the lamina propria is in the wavelength range. Finally, the fractal dimension of the binary image is calculated using Eq. (1).

### Five Spectrum-Based Methods for Cell Discrimination

When the oral dysplasia arises from the epithelial tissue, the number of nuclei in the basal-cell layer increases and the nuclear shape and size change [3, 4]. Moreover, according to previous studies [13–15], the nuclear spectrum presents a quantitative difference between normal, precancerous, and cancerous cells. Therefore, it is hypothesized that normal and cancerous cells differ in the nuclear spectrum of the basal-cell layer of epithelial tissue. In this work, the analyzed spectral data are obtained from two fluorescence images (obtained with F1 and F2 excitation, respectively). We choose the nuclei with well dyed and exclude the nuclei of border. Before analysis, the dark noise must be removed. The calibration formula is *F(λ)* = *I*_*F*_*(λ)*–*I*_*dark*_*(λ)*, where *F(λ)* denotes the fluorescent emission intensity of each pixel in the fluorescence image, *I*_*F*_*(λ)* denotes the spectral intensity of raw data of each pixel in the fluorescence image, and *I*_*dark*_*(λ)* denotes the spectral intensity of each pixel in the dark field. After the noise is removed, all of the nuclei in the basal-cell layer are chosen. Each nucleus comprises nine pixels. For each nucleus, the fluorescent emission spectral intensity is the average spectral intensity of the nine pixels.

According to the characteristics of the emission spectral shape, normal and cancerous cells were distinguished using five methods (three methods for spectrum obtained with F1 excitation and two methods for spectrum obtained with F2 excitation). The emission spectral shape for F1 excitation has two peaks and one valley (Fig. 3a); peak 1 at 560 nm, peak 2 at 705 nm, and valley at 630 nm). Since the normal cell and cancerous cell had different peak ratios or different valley values, the first method uses the peak ratio (PR), peak 1/peak 2, as a characteristic of spectral shape. The second method uses the peak and valley ratio (PVR), with the spectrum normalized by the intensity of peak 1. Then, the formula (peak1 × peak2)/valley is used as a characteristic value of spectral shape. The third method uses the area under the spectral curve (AUS1) normalized by the intensity of peak 1 as a characteristic of spectral shape. The emission spectral shape for F2 excitation has only one peak (Fig. 3b); peak at 560 nm). The fourth method uses the area under the spectral curve (AUS2) normalized by the intensity of peak as a characteristic of spectral shape. The fifth method uses the full width at half maximum (FWHM) of the spectral curve as a characteristic of spectral shape.

Each cell can obtain one characteristic value from each method. For each patient (two biopsies) and each method, the characteristic values of normal cells and cancerous cells were plotted as two distribution groups. The cut-off point and optimal performance (sensitivity and specificity) of each method were determined using the receiver operating characteristic (ROC) curve [23]. Sensitivity and specificity measure the inherent validity of a diagnostic method for dichotomous results [24, 25]. Details on how to calculate of sensitivity and specificity can be found elsewhere [25]. Sensitivity measures the proportion of actual positives that are correctly identified as positive, that is, the percentage of cancerous cells that are correctly identified. Specificity measures the proportion of negatives that are correctly identified, that is, the percentage of normal cells correctly identified. However, specificity and sensitivity rely on the cut-off point utilized to define “positive” and “negative” test results. Notably, sensitivity and specificity shift when the cut-off point shifts. The ROC curve delineates the trade-off between the sensitivity and (1-specificity) across a series of cut-off points [24].

### Spectrum-Based Cocktail Approach for Cell Discrimination

This section describes how the cocktail approach determines which spectral methods are effective for each patient. Previous works [15, 21, 22] have established that the lesion site, tumor size, age, and lymph node metastasis affected the cell spectrum. Therefore, in this work, patient data were sorted according to the age, lesion site, size or direct extent of the primary tumor (T), and degree of spread to regional lymph nodes (N). Here, a method with a mean sensitivity and a mean specificity of higher than 80 % was defined as effective with respect to a specific condition. For example, the ages of three patients (patients 18, 25, and 28) ranged from 30 to 39 years old. The mean sensitivity and mean specificity of the PR method for the three patients were 76.8 and 73.46 %, respectively (Table 2). Hence, the PR method is infeasible for patients in this age range. In contrast, the mean sensitivity and mean specificity of the PVR method were 91.43 and 83.6 %, respectively, for these patients, and thus the method was considered effective. Once the most effective methods were determined for each condition, their combination was used to diagnose a biopsy, depending on its conditions. The cell must undergo screening of all of the effective methods of the sample, followed by its diagnosis as a normal or cancerous cell. Figure 4 (in the Sect. 3.2) shows the flowchart that describes how the cocktail approach finds the effective methods for each condition.

### Combined Diagnosis Based on Fractal Dimension and the Cocktail Approach

The fractal dimension was first used to determine whether the biopsy tissue was normal or abnormal. The cocktail approach can further determine whether the cells were normal or cancerous. The biopsy was thus determined as normal or cancerous.

## Results and Discussion

### Morphological Identification Between Normal and Abnormal Tissues

**Fig. 2:**
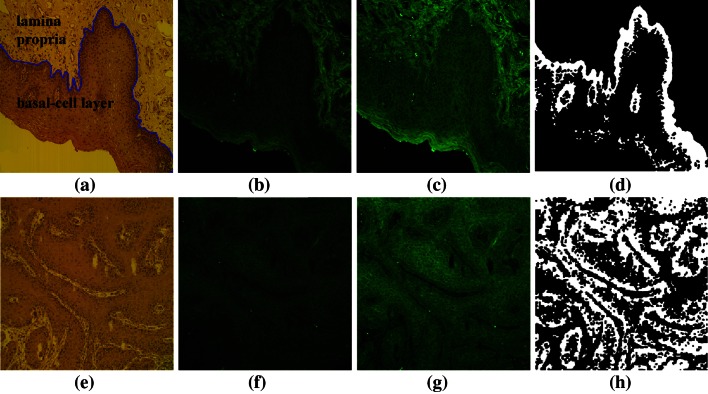
Biopsy image of patient 7. **a** Transmission image of normal tissue. Fluorescence images of normal tissue under (**b)** F1 and (**c)** F2 excitation. **d** Binary image of normal tissue for calculating fraction dimension. **e** Transmission image of cancerous tissue. Fluorescence images of cancerous tissue under (**f**) F1 and (**g**) F2 excitation. **h** Binary image of cancerous tissue for calculating fraction dimension

Table 1 shows information on the biopsies. Figure 2 displays the biopsy image of patient 7. Pathologists can obtain more information about the biopsy (e.g., the differentiation and the stage) from the transmission images (Fig. 2a, e).

**Table 1:** Patient information

Group	Patient no.	Age	Site	T	N	Stage	Number of nuclei	
							Normal	Cancer
Training	1	71	Tongue	2	2	IV	555	478
	2	50	Tongue	2	2	IV	158	156
	3	41	Mucosa	1	0	I	260	164
	4	42	Gum	2	2	IV	250	200
	5	44	Gum	1	0	I	102	200
	6	52	Gum	4	2	IV	166	160
	7	64	Tongue	1	0	I	360	400
	8	67	Mucosa	3	0	III	200	179
	9	51	Pyriform sinus	4	2	IV	133	578
	10	56	Gum	2	0	II	333	299
	11	47	Pyriform sinus	3	2	III	255	399
	12	51	Tongue	2	0	II	209	108
	13	56	Mucosa	3	0	III	300	367
	14	63	Palate	2	0	II	432	333
	15	50	Tongue	2	2	IV	589	601
	16	57	Gum	4	2	IV	200	309
	17	52	Tongue	2	0	II	378	678
	18	37	Tongue	1	2	IV	409	561
	19	78	Tongue	2	0	II	444	500
	20	60	Tongue	2	2	IV	400	266
	21	80	Tongue	1	0	I	289	260
	22	52	Palate	2	0	II	178	256
	23	65	Mucosa	2	0	II	300	457
	24	60	Tongue	3	0	III	398	457
	25	34	Tongue	1	0	I	405	302
	26	43	Tongue	2	2	IV	298	390
	27	61	Palate	4	0	IV	203	599
	28	32	Mucosa	1	0	I	592	212
	29	56	Mucosa	1	0	I	402	700
Testing	30	65	Mucosa	2	0	N/A	214	692
	31	41	Tongue	1	0	N/A	299	401
	32	31	Mucosa	1	0	N/A	272	459
	33	48	Tongue	2	3	N/A	393	598
	34	37	Mucosa	2	0	N/A	505	496

The number of nuclei are those in the basal-cell layer

*T T*the size or direct extent of the primary tumor, *T1, T2, T3, and T4* The size and/or extension of the primary tumor, *N* The degree of spread to regional lymph nodes, *N0* The tumor cells are absent from regional lymph nodes, *N1* The regional lymph node metastasis is present, *N2* The tumor has spread to an extent between N1 and N3, *N3* The tumor has spread to more distant or numerous regional lymph nodes

In this work, the complexity of the border between the basal-cell layer and the lamina propria was represented using the fractal dimension (Fig. 2a). Normal tissues had a clear border between the basal-cell layer and the lamina propria. In abnormal tissues, the cells eroded other cells, leading to a disordered border [4]. Figures 2d and 2(h) show the binary images. The white area of the binary image represents the border. Although the white area in the normal tissue is a continuous curve, the white area in the abnormal tissue is made up of discontinuous curves and spread over the entire image. Therefore, in the binary image, the border of abnormal tissue was more complex than that normal tissue. When the binary image contained a large number of white areas, the fractal dimension of the binary image was high. Hence, the fractal dimension of abnormal tissue was higher than that of normal tissue. Closely examining column 1 of Table 2 reveals that the criterion of fractal dimension for discrimination between normal and abnormal tissues is 1.73; below this value, the tissue was diagnosed as normal; otherwise, the tissue was diagnosed as abnormal. Notably, the chosen nuclei were well dyed and circle. Besides, when the objective power was altered, the fractal dimension changed. The change of fractal dimension was relative to the border complexity of magnified tissue. Moreover, the fractal dimension was high when the binary image of magnified tissue included a significant number of white areas.

**Table 2:** Fractal dimension value of normal and cancer tissues

No.	1		2			3			4			5			6			7	
	FD		PR			PVR			AUS1			AUS2			FWHM			Combination	
	Nor	Can	Sn.	Sp.	Cpoint	Sn.	Sp.	Cpoint	Sn.	Sp.	Cpoint	Sn.	Sp.	Cpoint	Sn.	Sp.	Cpoint	Sn.	Sp.
1	1.73	1.90	89.2	81.5	1.45	61.2	69.3	1.46	84.5	84.6	285	64.4	56.9	133	75.3	81.6	67	91.7	85.4
2	1.72	1.82	83.5	87.2	1.52	78.5	60.3	1.51	81.3	92.1	275	74.4	49.5	144	78.9	45.8	64	88.3	80.3
3	1.57	1.88	98	90.1	1.94	98.9	90.1	1.71	82.5	70.1	303	81.1	73.9	130	82.2	70.1	57.5	96.4	88.2
4	1.69	1.78	60.1	72.4	1.65	49.8	70.1	2.21	84.2	80.3	234	66.2	78.1	126	82.5	98.3	50.8	82.3	97.6
5	1.24	1.80	80.1	68.2	2.01	49.8	80.3	1.68	67.5	78.4	219	98	98.5	151	96.5	94.3	77	96.5	95.3
6	1.65	1.85	79.6	56.4	1.45	80.2	50.1	1.98	89.6	90.1	235	68.7	71.9	120	66.5	51.2	60.7	95.1	93.2
7	1.66	1.97	77.9	68.5	1.68	92.3	88.9	1.09	79.6	77.5	211	80.3	71.4	110	89.2	80.3	42	86.3	83.4
8	1.68	1.85	70.3	67.9	2.27	80.3	77.5	1.33	88.6	87.3	203	72.3	46.4	124	76.5	80.3	70	90.3	90.1
9	1.59	1.89	66.7	59.1	2.19	67.1	77.2	1.54	40.8	65.1	203	94.3	92.3	130	91.6	88.2	73	95.4	89.6
10	1.66	1.86	81.3	80.5	2.82	70.2	68.5	1.9	79.1	66.4	186	70.2	65.2	114	70.1	50.3	58.3	84.2	80.2
11	1.5	1.76	79.1	67.4	2.67	82.1	80.1	1.44	45.2	77.1	199	81.6	86.7	122	83.5	70.1	71	80.2	87.4
12	1.58	1.91	82.1	80.4	1.38	79.4	67.1	1.75	87.6	85.3	262	77.6	58.9	122	81.3	70.5	55.3	88.5	82.1
13	1.34	1.92	89.1	50.6	1.62	88.3	80.1	1.88	88.1	78.4	222	78.3	45.2	156	97.1	99.3	56	92.3	80.2
14	1.26	1.87	89.3	70.1	1.73	80.2	84.3	1.45	79.2	62.1	256	88.5	49.7	145	80.2	81.2	76	89.5	90.1
15	1.35	1.86	80.1	85.8	1.48	72.3	68.4	1.60	88.3	90.1	292	80.1	42.3	166	99	92.1	72	96.3	89.1
16	1.56	1.82	79.4	40.5	2.33	68.3	67.3	1.45	80.2	94.1	222	98.3	88.4	187	70.3	68.5	66	91.2	80.1
17	1.67	1.93	80.2	60.1	1.67	92.3	91.4	1.43	88.1	89.6	201	40.5	40.1	131	39.6	76.4	58	86.3	88.9
18	1.62	1.82	79.2	50.1	1.31	90.7	88.6	1.55	90.3	87.2	254	88.3	66	143	90.2	96.4	70	92.3	94.5
19	1.65	1.86	70.2	78.3	1.58	88.3	90.2	1.38	89.4	90.2	223	80.2	81.4	129	88.2	78	60	92.1	88.5
20	1.34	1.90	88.1	79.3	1.41	50.2	50.6	1.43	84.3	83.2	234	90.1	59.1	156	90.2	80.4	62	88.2	89.4
21	1.23	1.91	60.1	84.2	1.91	56.4	87.4	1.33	88.6	90.2	200	56.4	67.3	133	80.7	82.3	55	90.3	80.2
22	1.32	1.95	87.3	89.2	1.21	70.2	78.5	1.67	70.2	74.5	224	60.3	59.7	145	80.2	38.5	78	85.4	88.5
23	1.72	1.78	50.2	71.3	1.23	80.2	89.4	1.45	55.4	68.3	267	79.1	70.2	165	69.3	80.1	80	86.2	80.1
24	1.56	1.80	85.3	92.1	1.58	95.6	88.3	1.49	80.1	80.1	256	77.5	80.2	158	78.1	89.1	68	90.1	92.3
25	1.45	1.82	67.1	80.3	1.78	88.4	70.1	1.23	87.2	90.1	203	88.2	89.3	129	90.1	65.1	48	89.1	90.2
26	1.33	1.84	80.3	98.2	1.21	88.3	89.4	1.44	99.4	98.3	278	80.2	88.1	162	77.1	80.2	72	90.4	93.1
27	1.56	1.87	80.1	81.2	1.24	49.1	44.3	1.11	30.4	99.2	245	50.1	82.1	121	74.1	86.2	66	85.3	84.3
28	1.67	1.97	84.1	90	2.34	95.2	92.1	1.23	80.2	80.1	234	80.1	80	145	78.4	72.1	48	99.7	93.2
29	1.56	1.95	88.3	85.2	1.72	90.2	97.2	1.09	87.3	87.5	241	86.9	89.3	111	49.2	77.1	56	90.2	91.4
Mean	**1.533**	**1.866**	**78.3**	**74.7**	**1.74**	**77**	**77.1**	**1.51**	**78.5**	**82.7**	**236.8**	**76.9**	**69.9**	**138.2**	**79.5**	**76.6**	**63.4**	**90**	**87.8**
STD	**0.1608**	**0.058**	**10.36**	**14.14**	**0.43**	**15.25**	**13.83**	**0.26**	**16.08**	**9.78**	**285**	**13.52**	**16.68**	**18.76**	**12.95**	**15.28**	**9.84**	**4.53**	**5.21**

Sensitivity, specificity, and cut-off point of spectrum-based methods for distinguishing normal and cancer cells of 29 patients for training

*AUS1* area under spectral curve normalized by the intensity of peak 1, *AUS2* area under spectral curve normalized by the intensity of peak 2, *FD* fractal dimension, *FWHM* full width at half maximum of spectral curve, *PR* peak ratio, *PVR* peak and valley ratio, *Sn* sensitivity, *Sp*. specificity, *Cpoint* cut-off point of each sample, *STD* standard deviation

### Spectral Identification Between Normal and Cancerous Cells

**Fig. 3:**
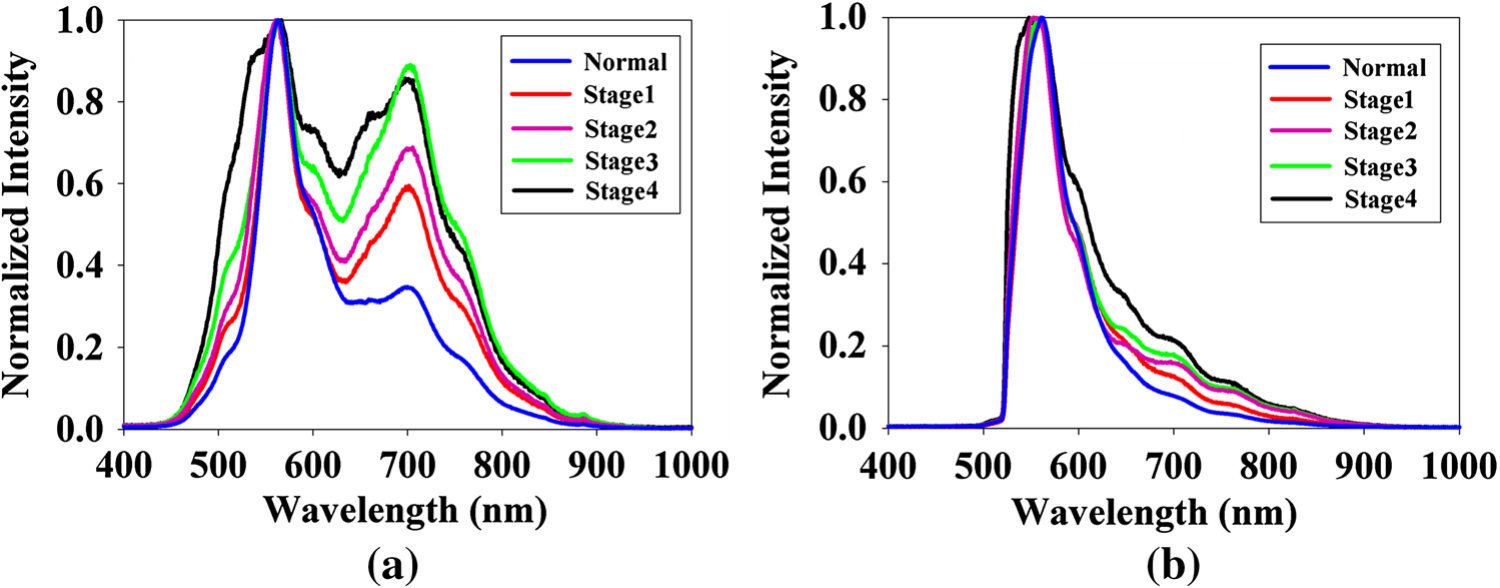
Difference of mean fluorescent emission spectrum of cells between normal and various oral cancer stages on tongue (patients 7, 12, 24, and 26). Mean fluorescence emission spectra of normal cells and cancer cells under (**a**) F1 and (**b**) F2 excitation

Since the ERL-MHIS provides the fluorescence spectrum of cell nuclei from the fluorescence images (Fig. 2b, c, f, and g), this work attempted to determine whether there was any spectrum-based difference between normal and cancerous cells in the basal-cell layer. Previous works [11–13] have established that the nuclear spectra at normal, precancerous, and cancerous stages are different. Hence, in this work, the nuclear spectrum was used to represent the spectrum of each cell. Figure 3 displays the typical cell spectra of various cancer stages, as obtained by the ERL-MHIS. Under F1 excitation (330–385 nm), the shape of the fluorescent emission spectrum had two peaks and one valley. Peak 1 was located at 560 nm and peak 2 was located at 705 nm. The valley was located at 630 nm. When the peak 1 intensity was normalized, the peak 2 intensity showed a difference between stages. Furthermore, the peak 2/peak 1 intensity ratio of normal cells was lower than that of cancerous cells. This finding is consistent with that of Roblyer et al. [26]. The peak 2 difference between normal and cancerous cells was due to the difference in porphyrin concentrations [27, 28]. Ramanujam et al. attributed the valley difference to cell metabolism. Under F2 excitation (470–490 nm), the fluorescent emission spectrum had only one peak, located at 560 nm. When the peak was normalized, the stages differed in the FWHM of the spectrum [27]. The difference can be used to monitor the changes of FAD concentration [29].

In order to distinguish between normal and cancerous cells in terms of spectral difference, the characteristics of spectral shape were described using five methods: PR, PVR, AUS1, AUS2, and FWHM. The performance of each method was evaluated based on the ROC curve. The cut-off point, sensitivity, and specificity were also determined (Table 2). Notably, under F1 excitation, although the normal cells of each patient had similar fluorescent emission shapes, the 29 patients differed in intensity of the two peaks or the valley. Under F2 excitation, these patients slightly differed in the FWHM of normal cells. The cancerous cells exhibited the same phenomenon under both excitations. Therefore, the diagnostic performances of the five spectral methods were not ideal. The methods were suitable for some patients, but not others. For example, the PR method yielded good results for patient 3, but not for patient 9. Hence, the five methods all showed high standard deviations.

**Fig. 4:**
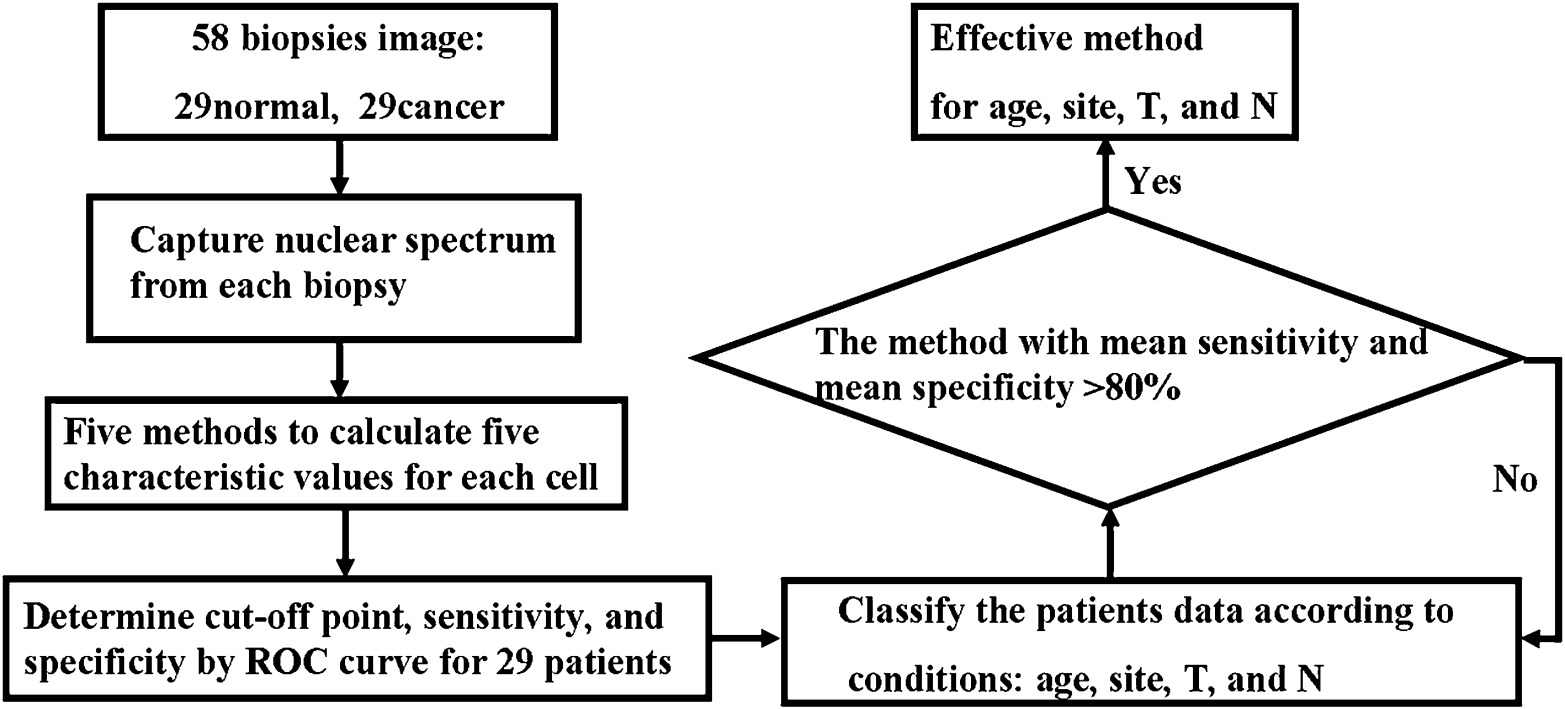
Flow chart of combination approach determining most effective methods

To solve the difference of nuclear spectrum between patients, this work classified the 29 sample data according to lesion site, tumor size, age, and lymph node metastasis. Then, based on the proposed cocktail approach, the effectiveness of the spectral methods in correlating with the sampling conditions was determined. Figure 4 shows how the cocktail approach determines the most effective methods for each sampling condition. Table 3 lists the most effective methods for each sampling condition. These methods can be combined according to the sampling conditions to diagnose a sample. For example, the combination of methods AUS1 and FWHM was used to diagnose sample 1 (71 years old, lesion on tongue, T2, and N2).

**Table 3:** Correlation between effective methods and patient’s conditions

Age	30–39	40–49	50–59	60–69	70–80
Method	PVR (1.34), AUS1 (230.3)	AUS2 (138.2), FWHM (65.7)	AUS1 (233)	FWHM (66.3)	AUS1 (236), FWHM (60.7)
Site	Tongue	Mucosa	Gum	Palate	Pyriform sinus
Method	AUS1 (242.6)	PVR (1.45)	AUS1 (219.2), AUS2 (139.6)	PVR (1.41)	AUS2 (126)
T	T1	T2	T3	T4	
Method	PVR (1.36), AUS1 (233.1)	AUS1 (247.5)	PVR (1.54), FWHM (66.3)	AUS2 (139.5)	
N	N0	N2			
Method	PVR (1.46)	AUS1 (246.5)			

Number in parentheses is optimal cut-off point of each effect method under different condition

*T* The size or direct extent of the primary tumor, *T1, T2, T3, and T4* The size and/or extension of the primary tumor, *N* The degree of spread to regional lymph nodes. *N0* The tumor cells absent from regional lymph nodes, *N1* The regional lymph node metastasis present, *N2* The tumor spreads to an extent between N1 and N3, *N3* The tumor spreads to more distant or numerous regional lymph nodes

Before a cell is diagnosed using the most effective method, the optimal cut-off point of the method must be determined. The optimal cut-off point was defined as the mean of total cut-off points under a specific condition. For example, for patients 18, 25, and 28 (age: 30–39 years), PVR was the most effective method. The optimal cut-off point for PVR under this condition was the mean of the three patients’ PVR cut-off points (1.55, 1.23, and 1.23, in Table 2). Moreover, each effective method had different optimal cut-off points under different conditions, because each condition had a different patient group. For example, the patient groups of T1 and T2 were different. Therefore, the optimal cut-off points of AUS1 differed for T1 and T2.

### Combined Diagnosis

The morphological changes of tissue were diagnosed using the fractal dimension. Although this method can diagnose the abnormal tissue, the abnormal tissue did not represent cancerous tissue. It may represent hyperplasia. In hyperplasia tissue, the number of cells increases and the border between the basal-cell layer and lamina propria becomes disordered [4]. Hence, in this work, the fractal dimension of hyperplasia tissue differed from that of normal tissue. Moreover, whether the cells were cancerous or normal was further confirmed using the cocktail approach.

**Fig. 5:**
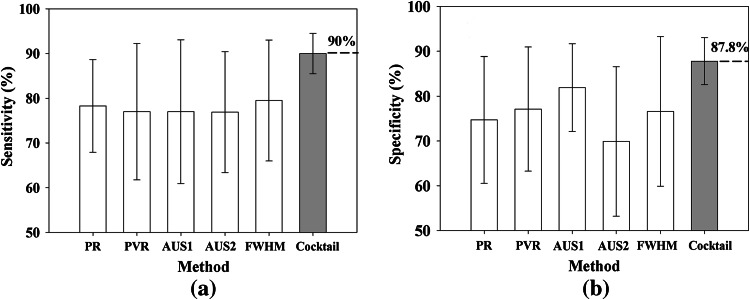
Comparison of performance of all methods. (**a**) Mean sensitivity and (**b**) mean specificity of 29 patients for each method

Figure 5 compares the performances of all methods. Because each method was appropriate for only some patients, the standard deviation of each method was large. In contrast, the cocktail approach showed a high mean specificity, high mean sensitivity, and small standard deviation, implying its better correlation with sample data. In addition, the fourteen patients with early stage oral carcinoma were successfully diagnosed with a sensitivity of 90 ± 4.65 % and a specificity of 87.2 ± 5.06 %. Moreover, 10 test samples were utilized to validate the training results (Table 3). Note that the type of sample (normal or cancerous) for the 10 test samples was not revealed to the analyst during their analysis of the samples. The 10 test samples were diagnosed according to the patient’s conditions. The sensitivity and specificity of the 10 test samples were 80.16 ± 4.5 % and 81.74 ± 2.26 %, respectively. Notably, the testing nuclei were chosen from well dyed cells and cells away border. The results can be enhanced by the correct patient’s condition. The concentration and the time of the H&E would be the key conditions.

In clinical application, the proposed approach can help pathologist quantitatively diagnose biopsies. The procedure of sample preparation and examination is the same as that for pathological examination in a clinic. The procedure of the diagnostic approach is controlled using programming. Therefore, the approach is convenient for pathologists. In addition, this is the first time that the ERL-MHIS was utilized to diagnose oral carcinoma biopsy. This study also provides a categorical approach of cellular spectrum to enhance diagnosis performance for clinical oral carcinoma research. The cut-off point of each method can be used as reference data by researchers. Moreover, this study proves that the spectrum of oral carcinoma cell relates to not only the cancer stage but also the patient’s conditions.

## Conclusion

This work developed morphological and spectral methods and then combined them to help pathologists diagnose oral carcinoma biopsy quantitatively. 68 biopsies of 34 oral carcinoma patients were diagnosed based on the ERL-MHIS. This is the first work to apply the ERL-MHIS to the cytopathological examination of oral carcinoma. The fractal dimension algorithm is applied to discriminate between normal and abnormal tissues in terms of morphological differences. For the spectral discrimination, normal and cancerous cells are distinguished using five methods. The spectra of normal and cancerous cells vary with patient. The diagnostic results of the five methods are thus not ideal. Therefore, the proposed cocktail approach is utilized to determine the effectiveness of spectral methods in correlating with the patient’s conditions. A combination of effective spectral methods that depends on the patient’s conditions is then used for diagnosing a biopsy. In addition to promoting the mean sensitivity and mean specificity, the proposed cocktail approach reduces the standard deviation. Moreover, this study successfully diagnosed oral carcinoma in its early stage. In the future, the k-nearest neighbor method or principle component analysis method will be used for finding characteristic molecules of different carcinoma stages. Furthermore, light-emitting diodes can be used as the light source of the ERL-MHIS to reduce scanning time.
